# Supplementary material for: Patients Treated for HCV Infection and Listed for Liver Transplantation in a French Multicenter Study: What Happens at Five Years?
Source: Viruses. 2022 Dec 31;15(1):137. doi: 10.3390/v15010137 (PMC9865729; doi:10.3390/v15010137)
Supplement: Supplementary file 1 [file viruses-15-00137-s001.zip › viruses-2129746-supplementary.pdf]

**Table S1: HCV genotype and HCV treatment**

| | Overall<br><br>n=179 (%) | Decompensated cirrhosis<br><br>n=75 (%) | HCC<br><br>n=104 (%) |
|---|---|---|---|
| HCV Genotype | | | |
| 1 | 102 (57.3) | 45 (60.0) | 57 (55.3) |
| 2 | 7 (3.9) | 5 (6.7) | 2 (1.9) |
| 3 | 41 (23.0) | 12 (16.0) | 29 (28.2) |
| 4 | 26 (14.6) | 12 (16.0) | 14 (13.6) |
| 5 | 2 (1.1) | 1 (1.3) | 1 (1.0) |
| Previous HCV treatment | 133 (74.3) | 55 (73.3) | 78 (75.0) |
| Current HCV treatment | | | |
| Sofosbuvir | 179 (100.0) | 75 (100) | 104 (100) |
| Simeprevir | 11 (6.2) | 2 (2.7) | 9 (8.7) |
| Daclatasvir | 111 (62.0) | 47 (62.7) | 64 (61.5) |
| Ledispasvir | 18 (10.1) | 10 (13.3) | 8 (7.7) |
| Ribavirin | 96 (53.6) | 38 (50.7) | 58 (55.8) |

*HCV: hepatitis C virus; HCC: hepatocellular carcinoma*

**Table S2: Serious adverse events under DAA therapy for HCV**

| SAEs | n |
|---|---|
| Infection | 9 |
| Decompensation of ascites | 1 |
| Anemia | 4 |
| Variceal bleeding | 3 |
| Encephalopathy | 3 |
| Asthenia | 5 |
| Cramps | 2 |
| Psychological disorders | 1 |
| Bradycardia | 1 |
| Erythema | 1 |
| Pruritus | 1 |
| Not known | 3 |

*SAEs: Serious Adverse Events*

**Table S3: Prediction of delisting for an improvement in 75 patients listed for decompensated cirrhosis at baseline of HCV treatment**

| Scoring system | Cut-off point | Youden Index | Sensitivity(%) | Specificity(%) | PPV (%) | NPV (%) |
|---|---|---|---|---|---|---|
| **MELD** | 10 | 0.241 | 37 | 87 | 50 | 80 |
| | 11 | 0.185 | 42 | 76 | 38 | 79 |
| | 12 | 0.324 | 58 | 75 | 44 | 84 |
| | 13 | 0.357 | 68 | 67 | 42 | 86 |
| | 14 | 0.424 | 84 | 58 | 41 | 91 |
| | 15 | 0.440 | 90 | 55 | 41 | 94 |
| | 16 | 0.295 | 90 | 40 | 34 | 92 |
| | 17 | 0.311 | 95 | 36 | 34 | 95 |
| | 18 | 0.202 | 95 | 26 | 31 | 93 |
| | 19 | 0.147 | 95 | 20 | 29 | 92 |
| | 20 | 0.129 | 95 | 18 | 29 | 91 |
| | 21 | 0.111 | 95 | 16 | 28 | 90 |
| | 22 | 0.038 | 95 | 9 | 27 | 83 |
| | 23 | 0.002 | 95 | 6 | 26 | 75 |
| | 24 | 0.055 | 100 | 6 | 29 | 100 |
| **Child-Pugh** | 5 | 0.121 | 21 | 91 | 44 | 77 |
| | 6 | 0.226 | 37 | 86 | 47 | 80 |
| | 7 | 0.416 | 68 | 73 | 46 | 87 |
| | 8 | 0.343 | 79 | 55 | 38 | 89 |
| | 9 | 0.324 | 84 | 48 | 36 | 90 |
| | 10 | 0.215 | 95 | 27 | 31 | 94 |
| | 11 | 0.161 | 100 | 16 | 29 | 100 |
| | 12 | 0.107 | 100 | 11 | 28 | 100 |
| | 13 | 0.018 | 100 | 2 | 26 | 100 |

*MELD: Model for End-Stage Liver Disease; PPV: positive predictive value; NPV: negative predictive value.*
